# Retrospective examination of pseudoprogression in *IDH* mutant gliomas

**DOI:** 10.1093/noajnl/vdad028

**Published:** 2023-03-18

**Authors:** Ethan A Wetzel, Matthew J Farrell, Blaine S C Eldred, Vicki Liu, Ishan Saha, Serendipity Zapanta Rinonos, Terry Prins, Tie Li, Minsong Cao, John Hegde, Tania Kaprealian, Negar Khanlou, Linda M Liau, Phioanh Leia Nghiemphu, Timothy Francis Cloughesy, Robert A Chong, Benjamin M Ellingson, Albert Lai

**Affiliations:** Department of Neurology, University of California, Los Angeles, Los Angeles, CA, USA; Department of Radiation Oncology, University of California, Los Angeles, Los Angeles, CA, USA; Department of Neurology, University of California, Los Angeles, Los Angeles, CA, USA; Department of Neurology, University of California, Los Angeles, Los Angeles, CA, USA; Department of Neurology, University of California, Los Angeles, Los Angeles, CA, USA; Department of Neurology, University of California, Los Angeles, Los Angeles, CA, USA; Department of Neurology, University of California, Los Angeles, Los Angeles, CA, USA; Department of Neurology, University of California, Los Angeles, Los Angeles, CA, USA; Department of Radiation Oncology, University of California, Los Angeles, Los Angeles, CA, USA; Department of Radiation Oncology, University of California, Los Angeles, Los Angeles, CA, USA; Department of Radiation Oncology, University of California, Los Angeles, Los Angeles, CA, USA; Department of Pathology and Laboratory Medicine, University of California, Los Angeles, Los Angeles, CA, USA; Department of Neurosurgery, University of California, Los Angeles, Los Angeles, CA, USA; Department of Neurology, University of California, Los Angeles, Los Angeles, CA, USA; Department of Neurology, University of California, Los Angeles, Los Angeles, CA, USA; Department of Neurology, University of California, Los Angeles, Los Angeles, CA, USA; Department of Neurosurgery, University of California, Los Angeles, Los Angeles, CA, USA; Department of Radiological Sciences, University of California, Los Angeles, Los Angeles, CA, USA; Department of Neurology, University of California, Los Angeles, Los Angeles, CA, USA

**Keywords:** contrast enhancement, glioma, *IDH*1/2, pseudoprogression, radiation necrosis

## Abstract

**Background:**

Tumor surveillance of isocitrate dehydrogenase (*IDH*) mutant gliomas is accomplished via serial contrast MRI. When new contrast enhancement (CEnew) is detected during postsurgical surveillance, clinicians must assess whether CEnew indicates pseudoprogression (PsP) or tumor progression (TP). PsP has been better studied in *IDH* wild-type glioblastoma but has not been well characterized in *IDH* mutant gliomas. We conducted a retrospective study evaluating the incidence, predictors, natural history, and survival of PsP patients in a large cohort of *IDH* mutant glioma patients treated at a single institution.

**Methods:**

We identified 587 *IDH* mutant glioma patients treated at UCLA. We directly inspected MRI images and radiology reports to identify CEnew and categorized CEnew into TP or PsP using MRI or histopathology.

**Results:**

Fifty-six percent of patients developed CEnew (326/587); of these, 92/326 patients (28% of CEnew; 16% of all) developed PsP and 179/326 (55%) developed TP. All PsP patients had prior radiation, chemotherapy, or chemoradiotherapy. PsP was associated with longer overall survival (OS) versus TP patients and similar OS versus no CEnew. PsP differs from TP based on earlier time of onset (median 5.8 vs 17.4 months from treatment, *P* < .0001) and MRI features that include punctate enhancement and enhancement location.

**Conclusion:**

PsP patients represented 28% of CEnew patients and 16% of all patients; PsP patients demonstrated superior outcomes to TP patients, and equivalent survival to patients without CEnew. PsP persists for <1 year, occurs after treatment, and differs from TP based on time of onset and radiographic features. Poor outcomes after CEnew are driven by TP.

**Key Points**

- PsP and true progression differ in time of onset and radiographic features for all glioma subtypes except grade 4 astrocytoma.
- PsP patients and patients who have not developed postsurgical new contrast enhancement have similar overall survival.
- PsP has similar characteristics in high- and low-grade *IDH* mutant gliomas.

**Importance of the Study**

New contrast enhancement (CEnew) during postsurgical glioma surveillance can be the initial indicator of tumor progression on MRI. However, not all instances of CEnew indicate true progression (TP), since some patients develop pseudoprogression (PsP) that initially appears to mimic TP but resolves or stabilizes in radiographic appearance on subsequent MRIs. Currently, differentiation between TP and PsP on MRI can only be accomplished retrospectively, and PsP has been poorly characterized in isocitrate dehydrogenase (*IDH*) mutant gliomas. Our study expands the understanding of PsP in *IDH* mutant glioma through analysis of a cohort of 587 patients with exclusively *IDH* mutant gliomas of all histologies. Fifty-six percent of patients developed CEnew and 28% of patients with CEnew exhibited PsP (16% of all patients). PsP occurs earlier than TP, persists for <1 year, and possesses unique radiographic features. PsP patients had improved survival compared with patients with TP and similar survival to patients without CEnew.

The current standard of care for gliomas includes surgical resection followed by radiation therapy (RT) and temozolomide (TMZ) or Procarbazine, Lomustine, and Vincristine (PCV) chemotherapy with or without an intervening period of active monitoring after surgery.^1^ Changes in patients’ treatment regimens are informed by the radiographic features seen on their MRI scans.^2^ New contrast enhancement on T1-weighted MRI (CEnew) can represent the first indication of progression.^2,3^ Alternatively, the possibility of pseudoprogression (PsP) has been increasingly recognized, where an instance of CEnew spontaneously resolves or remains radiographically stable on subsequent MRIs and does not represent progressive disease.^4–6^ However, real-time discrimination between these 2 possibilities is difficult and can only be accurately attempted retrospectively.

Isocitrate dehydrogenase (*IDH*)^6,7^ mutation status is a key genetic characteristic used to inform the prognosis and treatment of glioma patients.^3,4,5,7,8^ PsP affects both *IDH* mutant and wild-type glioma patients but has been poorly characterized in *IDH* mutant patients. Studies examining PsP in patients of mixed or unknown *IDH* status have reported the incidence rate of PsP to be between 6% and 31%.^4,9^ Recently, a study of a cohort of exclusively high-grade *IDH* mutant gliomas reported an incidence rate of 19%.^6^ Several studies have identified PsP as a positive survival prognosticator relative to true progression (TP) in patients of unknown *IDH* status,^10^ and mixed cohort studies have identified a higher rate of PsP in *IDH1* mutant grade 4 astrocytoma patients.^11^ Currently, there have been no studies to date examining PsP in a large cohort of exclusively *IDH* mutant patients that includes both low- and high-grade gliomas. Further investigation of the incidence, predictors, natural history, and prognostic impact of PsP will aid clinical decision-making when a clinician encounters CEnew on surveillance MRIs.

## Materials and Methods

### Patient Cohort

In this UCLA institutional review board approved study, in which informed patient consent was obtained prior to the collection and analysis of patient data, we retrospectively identified 724 *IDH* mutant diffuse glioma patients of all grades treated at UCLA between 1998 and 2021 (median follow-up = 5.23 years). We excluded 137 patients based on the following criteria: no reviewable MRI scans (*n* = 63), inadequate available scans (*n* = 72), or inability to apply 2016 WHO glioma classification due to lack of 1p19q codeletion status (*n* = 2).^12^ Of the 72 patients excluded for inadequate available scans, 65 patients had only 1–3 scans available for review and 7 patients were excluded for other reasons (eg, only preoperative imaging available [*n* = 1], large gaps in available scans [*n* = 6]). We did not attempt to apply 2021 WHO criteria, as many of the patients in our cohort lacked the necessary genetic testing to be adjusted to the WHO 2021 criteria (e.g. CDKN2A loss).^13^ However, in accordance with 2021 WHO guidelines, we are using the diagnosis “grade 4 astrocytoma (G4 Astro)” instead of glioblastoma (GBM). The resulting cohort of 587 patients is summarized in Table 1 and Supplementary Table S1.

**Table 1.** Demographic Characteristics of 587 *IDH* Mutant Patients in UCLA Cohort

	# of Patients	CEnew (%)	No CEnew (%)	PsP (%)	TP (%)
All	587	326 (56)	261 (44)	92 (16)	179 (30)
Male	345	199 (58)	146 (42)	55 (60)*	113 (63)*
Median age (y)	44	44	45	48	42
LO	148	55 (37)	93 (63)	16 (17)*	26 (15)*
AO	73	42 (58)	31 (42)	11 (12)*	25 (14)*
LA	139	77 (55)	62 (45)	22 (24)*	44 (25)*
AA	135	77 (57)	58 (43)	21 (23)*	39 (22)*
G4 Astro	92	75 (82)	17 (18)	22 (24)*	45 (25)*
*MGMT* methylated	190	96 (51)	94 (49)	36 (39)*	44 (25)*
*MGMT* unmethylated	119	61 (51)	58 (49)	14 (15)*	35 (20)*
Had RT	460	267 (58)	155 (34)	92 (100)*	169 (94)*
TMZ	400	273 (68)	127 (32)	75 (82)*	164 (92)*
Bevacizumab	124	122 (98)	2 (2)	15 (16)*	90 (50)*
GTR	233	110 (47)	123 (53)	32 (35)*	65 (36)*
STR	292	180 (62)	112 (38)	54 (58)*	94 (53)*
Biopsy	62	36 (58)	26 (42)	6 (7)*	21 (12)*

Percentages with a * in the PsP column are a percentage of all PsP patients (*n* = 92) and percentages with a * in the TP column are a percentage of all TP patients (*n* = 179). PsP and TP columns do not add up to 100%, as 55 patients with CEnew could not be placed into the PsP or TP group due to inadequate follow-up. AA, anaplastic astrocytomas; AO, anaplastic oligodendrogliomas; CEnew, new contrast enhancement; G4 Astro, grade 4 astrocytomas; GTR, gross total resection; *IDH*, isocitrate dehydrogenase; LA, low-grade astrocytomas; LO, low-grade oligodendrogliomas; PsP, pseudoprogression; RT, radiation therapy; STR, Subtotal Resection.

### Imaging Identification of Preoperative Enhancement, CEnew, and Separation Into PsP Versus True Progression

For each patient, we reviewed every available T1 MRI with and without contrast. MRI scans frequency varied between patients, though were typically conducted at 1-, 3-, or 6-month intervals. One-month intervals were most common after the detection of CEnew. CEnew and preoperative enhancement were identified by direct review of MRI imaging by the authors and confirmed by review of the radiology report. CEnew was defined as new contrast enhancement on postoperative scans that was not present on the previous scan. CEnew appearing on the immediate postsurgical scan was not included due to the high likelihood of capturing benign enhancement associated with surgical tissue damage. PsP was defined radiographically in 2 ways: the resolution of CEnew (*n* = 106 instances) or the persistence of radiographically stable contrast CEnew for >1 year (*n* = 13 instances). A CEnew lesion that initially grew but eventually stabilized or resolved in the absence of new treatment would also be called PsP. Although radiation necrosis is acknowledged as a subset of PsP,^4^ we did not distinguish radiation necrosis within our PsP patients.^4^ A single patient could have multiple CEnew instances arising synchronously or asynchronously, and all were captured in this study. Each CEnew instance was tracked until it was surgically resected, resolved, stabilized on imaging or the patient’s treatment was changed (generally after sustained increase in size). Patients who had yet to develop CEnew were designated “No CEnew (censored).” To place a patient in the No CEnew group, a minimum of 2 years of follow-up was required, where patients with less than 2 years of follow-up were censored based on last documented contact. CEnew that could not be tracked for at least 1 year were tracked through all available patient scans and categorized separately as “censored CEnew” if their fate (PsP vs TP) could not be resolved. Fifty-five patients had CEnew that was radiographically stable for <1 year and were designated as “censored CEnew.” RANO criteria were not strictly applied to differentiate PsP from TP due to the retrospective nature of this study, although the criteria applied generally follow RANO criteria.

To ensure that CEnew resolution was not due to treatment response, both conditions for PsP required the absence of initiation of radiation or chemotherapy and no sustained increase in the patient’s prescribed dose of corticosteroids. Sustained increase in corticosteroids was defined by an increase in corticosteroid dose that was initiated at the appearance of CEnew and maintained at subsequent scans. If these treatments, including corticosteroids, were initiated, the instance of CEnew was considered TP. However, PsP patients may have completed a treatment plan that was initiated prior to the appearance of CEnew and was continued without change (*n* = 62); Of these, 36 of the treated PsP patients received TMZ, 15 received bevacizumab, 3 received clinical trial drugs, 7 received CCNU, and 1 received carboplatin.

If there was a repeat resection (*n* = 227), CEnew was interpreted as TP if there was histopathological evidence of tumor in the enhancing region (*n* = 219). Surgical resection of enhancing tissue that was deemed recurrent tumor by the neuropathologists was considered TP, whereas resected tissue with Ki-67 <1%, no active mitoses, and no microvascular proliferation was deemed PsP (*n* = 8 instances). One hundred and four patients received bevacizumab after the appearance of CEnew, as this is a common treatment for radiation necrosis, and MRIs were tracked to ensure enhancement did not reappear during or within 1 year of completion of bevacizumab treatment to be deemed PsP patients (*n* = 6).

When determining radiographic predictors of PsP and TP, we retrospectively examined the following radiographic features by direct review of MRI images: periventricular location, defined as CEnew directly abutting the lateral ventricle; resection margin, defined as CEnew directly abutting the resection margin; band thickening, defined as the thickening of postsurgical enhancement of the rim of the resection cavity; punctate enhancement, defined as CEnew <1 cm in size; nodularity, defined as CEnew >1 cm in size; diffuse enhancement, defined as the presence of faint CEnew spanning >5 cm in largest dimension; synchronous, defined as 2 or more CEnew lesions that appeared on the same MRI scan; concurrent, defined as 2 or more CEnew lesions that were present on the same scan but did not first appear on the same scan; and serial, defined as 2 or more CEnew lesions that were not present on the same scan at any point.

### Pathological and Molecular Analysis

Board-certified neuropathologists from UCLA and pathologists from outside institutions where the surgeries were conducted assessed all tumor samples. Looking at the individual pathology reports, we followed the 2016 WHO classification criteria of CNS tumors when updating obsolete diagnoses such as low-grade mixed gliomas. We used either immunohistochemistry, polymerase chain reaction (PCR) sequencing, or next-generation sequencing (generally either from Strata or Foundation Medicine) to identify variants in *IDH1* or *IDH2* genes. *MGMT* gene promoter methylation assay was generally performed by LabCorp or NeoGenomics Laboratories using bisulfite modification of tumor deoxyribonucleic acid (DNA) and quantitative PCR to detect CpG methylation.

### Radiation Dosimetry Analysis

Dosimetric data were analyzed for 20 patients—the 10 most recently treated patients who went on to develop PsP and the 10 most recently treated patients who went on to develop TP. They received RT between 2016 and 2021. For each patient, the T1-weighted postcontrast sequence from the MRI showing CEnew was imported into the planning software, Medical Image Merge (MIM), and fused with the CT simulation scan and dosimetric data from the prior RT plan. The area of CEnew was contoured. Dosimetric data were extracted for the CEnew contour, including mean RT dose, and the structure was geometrically compared with the original planning target volume (PTV).

### Statistics

We generated Kaplan–Meier (KM) estimator curves and performed Cox regression analyses for both overall survival (OS) and residual OS to compare the outcomes of various patient groups. We defined OS as the time from initial surgical resection to the patient’s censored date of last contact or death. Residual OS was defined as the time between the appearance of PsP or TP until patient censoring or death. KM curves were also used to determine median time to time-based events (eg, Time to CEnew) and patients without that event were included as censored patients. All survival analyses and analysis of time-based events were performed in GraphPad Prism and categorical variables were analyzed using Fisher’s exact test. When comparing the PsP and TP groups, the PsP group included any patient with at least 1 instance of PsP, though these patients could have had a separate instance of TP. When quantifying the incidence of PsP, TP, CEnew, etc., we used the term estimated incidence, as we utilized a cohort including censored patients. As the outcome of censored patients is unknown, we have likely underreported the incidence, and thus refer to this as “estimated incidence.” When conducting exploratory analyses to determine the predictors of CEnew, PsP, and TP, we used a Cox regression model with the following covariates: age at intervention, gender, extent of resection, Karnofsky Performance Status (KPS) ≤70, preoperative enhancement (only in Supplementary Tables S4 and S5), CEnew (only in Supplementary Table S7), PsP (only in Supplementary Tables S9 and S11–S14), and TP (only in Supplementary Table S11).

## Results

### Incidence, Time of Onset, Predictors, and Survival Impact of CEnew

First, we sought to identify all instances of new contrast enhancement occurring postsurgery (CEnew) in our cohort of 587 *IDH* mutant patients. The 587-patient cohort included patients with low-grade oligodendrogliomas (LO) (*n* = 148), anaplastic oligodendrogliomas (AO) (*n* = 73), low-grade astrocytomas (LA) (*n* = 139), anaplastic astrocytomas (AA) (*n* = 135), and grade 4 astrocytomas (G4 Astro) (*n* = 92). We identified 326 patients with a total of 517 CEnew instances (Supplementary Table S2). For the 132 patients with multiple instances of CEnew, we focused on the first instance of CEnew to determine the time of onset, predictors, and survival impact of CEnew in our *IDH* mutant cohort. The estimated incidence of CEnew was 56% (326/587) in our entire cohort, and was highest for G4 Astro (75/92, 82%) and lowest for LO patients (55/148, 37%) (Table 1). As determined by KM analysis, the median time (months) from initial surgery to CEnew was: All = 42.6, LO = 79.1, AO = 48.4, LA = 37.0, AA = 26.7, and G4 Astro = 22.1 months (LO vs G4 Astro: *P* < .0001) (Supplementary Figure S1).

To identify predictors of CEnew, we performed Cox regression analysis separately on each subtype and found that gross total resection (GTR) in AO, AA, and G4 Astro is associated with decreased risk of CEnew (Table 2) (All: GTR: HR = 0.63, *P* = .12; AO: GTR: HR = 0.09, *P* = .03; AA: GTR: HR = 0.52, *P* = .008; G4 Astro: GTR: HR = 0.42, *P* = .04). In an additional analysis including only patients with known preoperative enhancement status (*n* = 433, Supplementary Table S3) the presence of preoperative enhancement was found to predict decreased time to CEnew in All and AA patients, as might be expected (All: HR = 1.39, *P* = .02; AA: HR = 2.09, *P* = .02) (Supplementary Table S4). By KM and Cox regression analysis, preoperative enhancement was associated with reduced OS in All, AO, and LA patients (Supplementary Figure S2; Supplementary Table S5). Preoperative enhancement was also shown to predict increased likelihood of developing CEnew in All, LO, AO, LA, and AA patients using Fischer’s exact *T*-test, though the proportions used include censored patients and cannot account for those who have yet to develop CEnew (Supplementary Table S6).

**Table 2.** Cox Regression Analysis Evaluating Predictors of Enhancement Free Survival (EFS) in All Patients, LO, AO, LA, AA, and G4 Astro Subgroups

Variable (EFS)	All			LO			AO			LA			AA			G4 Astro		
	HR	*P*	95% CI	HR	*P*	95% CI	HR	*P*	95% CI	HR	*P*	95% CI	HR	*P*	95% CI	HR	*P*	95% CI
Age at intervention	1.01	.04*	[1.00, 1.02]	0.99	.9	[0.97, 1.03]	1.02	.2	[0.99, 1.05]	1	.6	[0.98, 1.03]	1	.8	[0.98, 1.03]	1.01	.1	[0.99, 1.032]
Gender (F = ref)	1.1	.3	[0.90, 1.41]	0.98	.9	[0.57, 1.71]	0.52	.05*	[0.27, 1.00]	0.99	.9	[0.61, 1.61]	1.31	.3	[0.81, 2.14]	1.55	.09	[0.94, 2.56]
KPS ≤70	1.59	.03*	[1.04, 2.43]	3.09	.2	[0.63, 15.18]	1.17	.8	[0.44, 3.13]	0.73	.5	[0.29, 1.83]	1.95	.3	[0.52, 7.29]	1.75	.2	[0.81, 3.78]
GTR (STR/biopsy = ref)	0.63	.1	[0.49, 1.08]	0.79	.4	[0.38, 2.12]	0.16	.05*	[0.01, 0.80]	1.92	.9	[0.24, 1.12]	0.52	.008*	[0.319, 1.92]	0.36	.04*	[0.18, 0.97]
Events	326			55			42			72			77			75		

AA, anaplastic astrocytomas; AO, anaplastic oligodendrogliomas; G4 Astro, grade 4 astrocytomas; GTR, gross total resection; LA, low-grade astrocytomas; LO, low-grade oligodendrogliomas.

*in Table 2 indicates Statistical Significance (p <0.05) by Cox Regression Analysis.

**Figure 1.**
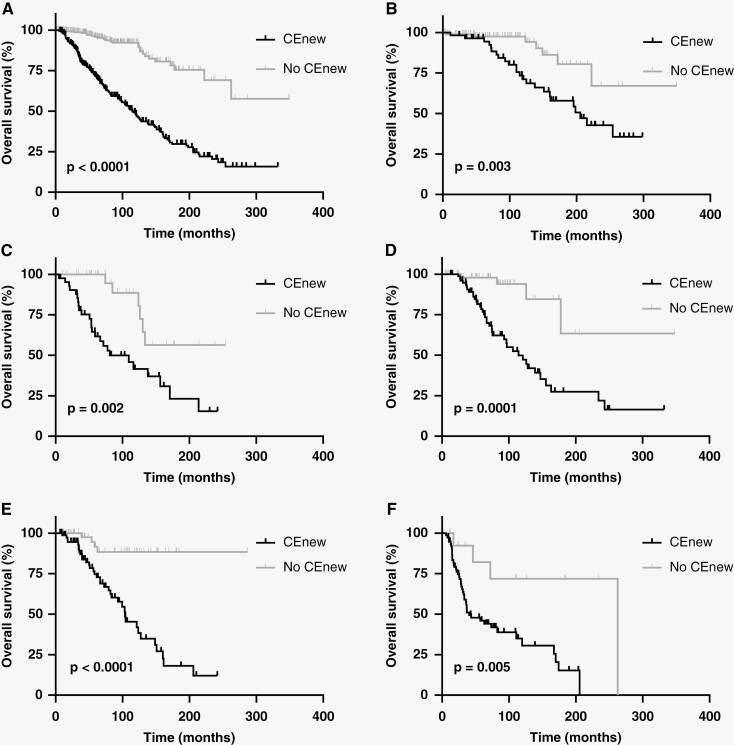
Prognostic impact of CEnew and preoperative enhancement. Kaplan–Meier analysis of UCLA *IDH* mutant glioma patients evaluating the prognostic impact of CEnew (A–F). (A–F) show OS for patients with CEnew and patients with No CEnew. Log-rank *P* values comparing median OS between No CEnew and CEnew for All (A), LO (B), AO (C), LA (D), AA (E), and G4 Astro (F) were <.0001, .003, .002, .0001, <.0001, and .005, respectively. AA, anaplastic astrocytomas; AO, anaplastic oligodendrogliomas; CEnew, new contrast enhancement; G4 Astro, grade 4 astrocytomas; *IDH*, isocitrate dehydrogenase; LA, low-grade astrocytomas; LO, low-grade oligodendrogliomas; OS, overall survival.

By KM analysis, the median OS for all patients who developed CEnew was 115 months versus undefined (not reached) for those without CEnew (*P* < .0001) (Figure 1A). Similar analysis by subtype showed that CEnew was associated with worse OS in all glioma subtypes (Figure 1B–F). In Cox regression analysis, CEnew was associated with decreased OS in All patients and all glioma subtypes (Supplementary Table S7).

#### PsP occurs in 28% of CEnew patients, differs from true progression in time of onset, and typically persists for less than 1 year

To investigate differences between PsP and TP, we categorized CEnew patients into PsP or TP groups as described in Methods. One hundred and nineteen unique instances of PsP were identified in 92 patients (92/326, 28% of patients with CEnew, and 16% of entire cohort), and 321 unique instances of TP were identified in 179 patients (179/326, 55% of patients with CEnew) (Table 1). The estimated incidence of PsP was highest in G4 Astro (32%) patients and lowest in LO (11%) (LO vs G4 Astro: *P* = .007) (Table 1). The estimated incidence of TP was also highest in G4 Astro patients (45/92, 49%) and lowest in LO (26, 18%) patients (*P* < .00001) (Table 1), although 79% of LO patients were censored versus 48% of G4 Astro patients. Of the 92 PsP patients, 89/92 (97%) had received RT (with or without chemotherapy) and 3/92 (3%) had received chemotherapy alone (Supplementary Table S8). Eighty-four percent of TP patients received prior RT or chemotherapy.

**Figure 2.**
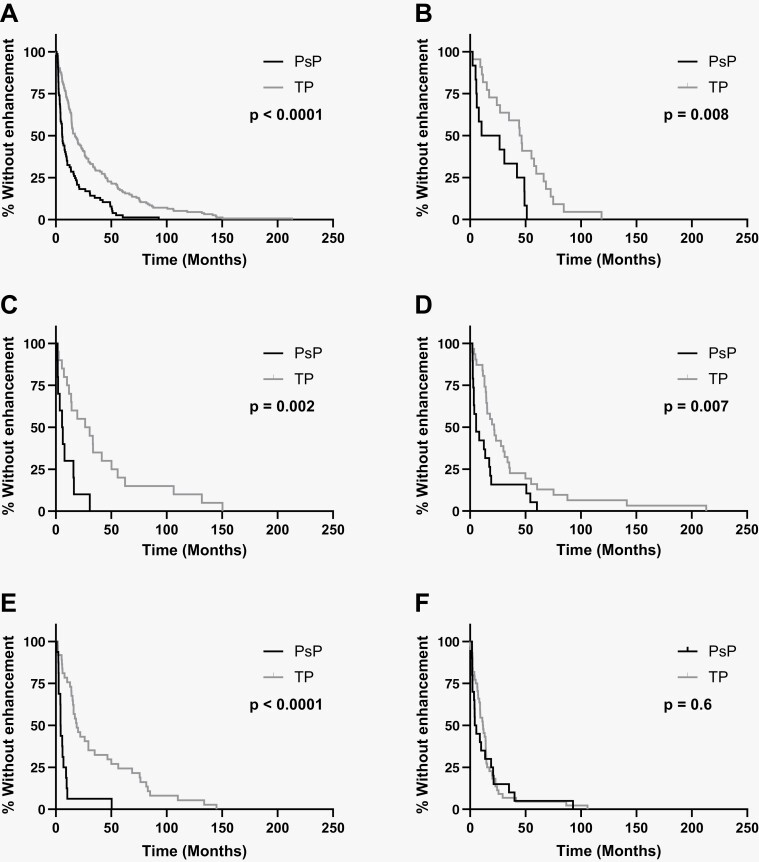
Time from definitive therapy to PsP or TP. Kaplan–Meier analysis of the time from definitive therapy (chemotherapy or radiation) initiation to the appearance of PsP or TP in All (A), LO (B), AO (C), LA (D), AA (E), and G4 Astro (F). Log-rank *P* values comparing the time to PsP versus TP in All, LO, AO, LA, AA, and G4 Astro were <.0001, .008, .02, .007, <.0001, and .6, respectively. AA, anaplastic astrocytomas; AO, anaplastic oligodendrogliomas; G4 Astro, grade 4 astrocytomas; LA, low-grade astrocytomas; LO, low-grade oligodendrogliomas; PsP, pseudoprogression.

In all PsP patients, the median time from initial surgery to a patient’s first instance of PsP was 20.7 months, compared with 37.1 months to the first instance of TP (Supplementary Figure S3). After stratification by subtype, initial PsP was found to appear earlier than initial TP in the AO, LA, and AA glioma subgroups (LO: *P* = .8; AO: *P* = .0002; LA: *P* = .03; AA: *P* < .0001; G4 Astro: *P* = .3) (Supplementary Figure S3). By multivariate analysis, PsP was also found to occur earlier than TP in All, AO, LA, and AA patient subtypes but not G4 Astro (Supplementary Table S9). Given that PsP appearance is always preceded by treatment (Supplementary Table S8) and not all *IDH* mutant patients begin definitive therapy immediately after initial surgery, we repeated this analysis using the time from the start of each patient’s first definitive therapy (chemotherapy or RT) to PsP and TP to examine a more clinically relevant time interval. Patients with TP that was not preceded by treatment (spontaneous TP) were removed. In all PsP patients, the median time to PsP from treatment was 5.8 months, compared with 17.4 months from treatment to TP (Figure 2; *P* < .0001). This difference was significant for all glioma subtypes except G4 Astro (All: PsP = 5.8, TP = 17.3, *P* < .0001; LO: PsP = 18.4, TP = 45.5, *P* = .008; AO: PsP = 6.0, TP = 28.4, *P* = .002; LA: PsP = 5.5, TP = 15.5, *P* = .007; AA: PsP = 4.3, TP = 26.1, *P* < .0001; G4 Astro: PsP = 4.9, TP = 11.7, *P* = .6). Overall, PsP is likely to occur within 6 months of a patient’s first therapy, except for LOs when PsP occurred at 18 months from therapy.

Of the 119 unique instances of PsP examined for the 92 patients, the median duration of PsP, from the appearance of contrast enhancement to its resolution, was 8.1 months by KM analysis (Supplementary Figure S3). The median duration of PsP spots by histopathological diagnosis was also examined and not found to be significantly different between subtypes, except between LA and AA (median [months]: LA = 9.6, AA = 6.3; *P* = .04) (Supplementary Figure S3), though no significant predictors of increased or decreased PsP duration were identified in multivariate analysis (Supplementary Table S10). In the 26 PsP patients with more than 1 instance of PsP, the median duration of first vs subsequent PsP instances was found to be similar except for fourth PsP in 2 patients (medians [months]: second PsP duration = 5.7 [*n* = 26], third PsP duration: 6.3 [*n* = 5], fourth PsP duration: 14.15 [*n* = 2]). Thirteen cases of PsP (11%) did not resolve but were radiographically stable for >1 year (Supplementary Figure S3) and were clinically indistinguishable.

#### PsP predicts improved OS versus TP patients and similar OS to patients without CEnew

**Figure 3.**
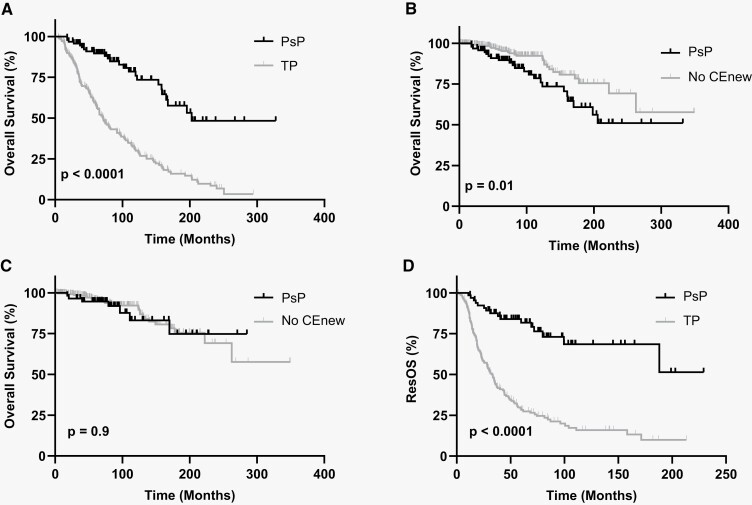
Prognostic impact of PsP on OS, Progression Free Survival (PFS), and resOS. Kaplan–Meier analysis examining the survival effects of PsP, TP, and No CEnew. (A) and (D) show OS and resOS, respectively, for patients with PsP and TP (log-rank *P* < .0001 and <.0001, respectively). (B) and (C) show OS for patients with PsP and no CEnew, where (C) has patients with both PsP and TP removed (log-rank *P* value = .01 and .9, respectively). CEnew, new contrast enhancement; OS, overall survival; PsP, pseudoprogression; resOS, residual overall survival.

Since several studies in the *IDH* wild-type glioma literature have shown an association between PsP and improved OS when compared with TP patients,^10,11,14^ we sought to investigate this possibility in our cohort of *IDH* mutant patients. The median OS for All patients was 170.7 months. The median OS for the PsP patients and TP patients was 202.8 and 71.5 months, respectively (*P* < .0001) (Figure 3A). KM analysis for each pathological subgroup revealed that PsP was associated with longer OS versus TP patients in all glioma subgroups (Supplementary Figure S4). Multivariate analysis showed that PsP is associated with increased OS in All patients, a combined LO + AO cohort (to achieve sufficient statistical power), and the AA cohort (All: HR = 0.42, *P* = .002; LO + AO: HR = 0.15, *P* = .03; AA: HR = 0.19, *P* = .01) (Supplementary Table S11). The hazard ratios of the LA and G4 Astro groups did not reach significance (Supplementary Table S11).

Next, we examined the residual survival of patients after the first instance of PsP or TP, where residual overall survival (resOS) was defined as the time between the appearance of PsP or TP to the patient’s date of death or censoring. For patients with both PsP and TP, patients were placed into the PsP or TP groups based on which occurred first. Using KM analysis, PsP was associated with longer resOS compared with the resOS for TP in All and all glioma subtypes (Figure 3D and Supplementary Figure S5). These results were confirmed by multivariate analysis in which PsP was also associated with increased resOS in All patients and all glioma subtypes (Supplementary Table S12).

In a study of GBM patients of unknown *IDH* status, OS was previously found to be greater in those with PsP than in patients who did not develop contrast enhancement.^14^ Therefore, we sought to examine the difference in OS between these 2 groups in *IDH* mutant glioma using KM analysis. PsP patients were found to perform worse than No CEnew patients in the All and LA subgroups and similar to No CEnew patients in the LO, AO, AA, and G4 Astro subgroups (Supplementary Figure S6). This was confirmed in multivariate analysis, where the No CEnew group performed better than the PsP group in the All and LA analyses but equivalent in the others (All: HR = 1.99, *P* = .02; LA: HR = 5.44, *P* = .01) (Supplementary Table S13). However, since 35 PsP patients also had an instance of TP (after PsP: *n* = 20, before PsP: *n* = 12), we removed these patients to derive a “pure” PsP group. Our analysis revealed that “pure” PsP patients without an instance of TP perform similar to patients without CEnew in terms of OS in All patients and when broken into glioma subgroups (Figure 3C and Supplementary Figure S7). This was also seen in multivariate analysis, where the pure PsP patients and No CEnew group had similar OS (Supplementary Table S14).

### Radiographic Characteristics of PsP and TP

To determine radiographic features distinguishing PsP versus TP, we characterized MRI features of 119 PsP and 318 TP spots (Supplementary Tables S15 and S16). We found that periventricular location and punctate size predicted PsP, while TP instances were often nodular and abutting the resection cavity. Twenty-three of the 119 (19%) instances of PsP were found in the periventricular region, while only 4 (1.2%) TP instances were periventricular (*P* < .0001 by Fisher’s exact *T*-test). Eleven (9%) instances of PsP were found to directly abut the margin of the resection cavity compared with 297 (93%) of TP instances (*P* < .0001). Fifty-six (47%) PsP instances were characterized as punctate compared with 4 (1.3%) TP instances (*P* < .0001). Twenty-two (18%) PsP instances were found to be nodular compared with 234 (74%) TP instances (*P* < .001). Several other radiographic features including thickening of enhancement along the resection margin, diffuse enhancement, etc. were analyzed as possible predictors of PsP and TP but were found to differ insignificantly (Supplementary Tables S15 and S16).

### Radiation Exposure in PsP and TP

In the 10 PsP and 10 TP cases for which radiation dosimetry data were analyzed, CEnew developed within the prior radiation field in most cases. The contoured area of enhancement fell completely within the PTV in 8 (80%) of the patients with PsP and 7 (70%) of those with TP (Supplementary Table S17). For the 5 cases in which some or all of the enhancement fell outside the PTV, the area of enhancement was still exposed to RT dose, with mean doses of 46 and 27 Gy in the PsP cases and 43, 61, and 61 Gy in the TP cases. We then examined the estimated incidence of PsP in patients who received re-irradiation. 64/587 patients in our cohort received re-irradiation, and 25% (16/64) developed PsP. Of these 16 patients, 10 had PsP after the first course and 6 had PsP after the second course. Of the 6 patients with PsP after re-irradiation, 4 patients received IMRT (median dose = 59.4 Gy; median number of fractions = 30) and 2 received stereotactic RT (median dose = 25 Gy; median number of fractions = 5, where 4 of the 6 had repeat RT in the same radiation field).

## Discussion

CEnew on T1-weighted MRI is recognized as a hallmark of tumor progression of *IDH* mutant gliomas, but it can also represent PsP, where an instance of CEnew does not represent progressive disease and spontaneously resolves or remains stable without treatment. However, critical, real-time determination of these 2 possibilities is difficult. PsP has been somewhat better characterized in *IDH* wild-type glioma, but only 1 study has examined PsP in an exclusively *IDH* mutant cohort.^6^ Our study examines PsP in a large cohort of *IDH* mutant patients that includes grade 2, 3, and 4 patients. Here, we sought to examine the incidence, characteristics, predictors, and survival implications of PsP to better inform clinicians’ management of CEnew, PsP, and TP. In doing so, we attempt to provide guidance for clinical navigation upon development of CEnew.

We retrospectively examined the contrast enhanced T1-weighted MRI imaging of a large cohort (*n* = 587) of exclusively *IDH* mutant glioma patients, identified all instances of CEnew, and determined the incidence, characteristics, and survival implications of CEnew, PsP, and TP. CEnew occurred in 56% of our *IDH* mutant cohort and predicted worse OS. Risk factors of CEnew include preoperative enhancement for AA patients and subtotal resection in AO, LA, and AA. PsP was found to be a transient event characterized by an incidence of 28% in CEnew patients, later onset than TP, punctate radiographic appearance often distant from the resection cavity, and a duration of <1 year, though 11% of PsP cases were persistent and remained radiographically stable. In terms of the effect of PsP on survival, PsP is associated with improved OS when compared with TP patients in the entire cohort, AAs, and LO + AOs. PsP is also associated with increased resOS, and PsP patients performed similarly in terms of OS to patients without CEnew.

The existing literature examining PsP in an exclusively *IDH* mutant cohort is limited, though 1 retrospective study exists that examined 200 high-grade *IDH* mutant patients.^6^ This study looks at a smaller cohort of patients compared with our current study, primarily because of the lack of low-grade gliomas. Both datasets include censored patients resulting in likely underestimated incidence rates. They reported a PsP incidence of 19% (*n* = 38) in their censored cohort and did not identify any significant differences between PsP incidence among high-grade pathologies.^6^ In our study, we report a similar estimated incidence rate of 16% and did not identify significant differences in PsP incidence within high- or low-grade pathologies. In terms of the time of PsP onset, the aforementioned study reported that PsP occurred within a median of 10.5 months from initial diagnosis and within 6 months of RT in 68% of patients. These results are consistent with our results, where we found the median time from RT or chemotherapy to PsP to be 5.8 months, which was similar in all histologic subgroups except LO with a median time after RT of 18.4 months. Interestingly, we report a longer interval from initial surgery to PsP of 20.7 months, though this is likely because our study included low-grade patients who may have had a period of monitoring before the initiation of definitive therapy. In terms of the radiographic characteristics of PsP, the existing study reported 37% of PsP patients had contrast enhancing lesions that did not resolve and remained radiographically stable for at least 12 months, and that this phenomenon occurred in a similar frequency among different glioma subtypes.^6^ We utilized the same 12-month criteria of radiographic stability to call a lesion PsP and observed this phenomenon in only 11% of patients with no significant difference between subtypes. Interestingly, our data further revealed that patients with stable CEnew for <3 months had similar OS to the PsP group (Supplementary Figure S8), indicating a cutoff of 3 months of stability may be sufficient to differentiate PsP from TP. In those that did resolve, the existing study reported a PsP duration of 6 months, similar to the 8-month interval reported here.^6^ Overall our results expand upon the previous study of PsP in IDH mutant gliomas by including low-grade patients and reveals that PsP has similar characteristics in low and high-grade IDH mutant gliomas.

The existing literature in *IDH* wild-type tumors and studies with a mixed cohort of mutant and wild-type patients have reported the incidence rate of PsP is 18% of all patients or 40% of patients with CEnew^15–17^ which is also similar to our reported PsP incidence of 16% of all patients and 28% of CEnew patients, though it is difficult to compare these values due to the varying definitions of PsP used between studies and use of censored patients. A study of 71 low-grade glioma patients of mixed *IDH* mutation status reported similar findings for PsP in terms of time of onset and duration, correlation with RT, and smaller size on MRI than TP.^9^ Several studies have reported PsP is associated with improved OS relative to TP in cohorts of mixed *IDH* status,^11,14,18,19^ and we observed this in our cohort of exclusively *IDH* mutant patients.

Previous studies have reported an association between *MGMT* promoter methylation and increased risk of PsP in a cohort of mixed *IDH* status patients.^14,16,17,20^ We did not identify any predictive relationship between PsP and *MGMT* promoter methylation, though this discrepancy may be reconciled by recent reports that *MGMT* promoter methylation has less utility as a predictor of OS in *IDH* mutant glioma patients.^21,22^ It was previously reported that G4 Astro patients with PsP outperformed patients without CEnew in terms of OS.^14^ Our results did not demonstrate this association within the G4 Astro subgroup or any other glioma subgroup.

Previous studies have reported that PsP is associated with RT,^18,23–26^ so we investigated this possibility in our cohort by examining time from definitive therapy to PsP and TP and the radiation dosimetry of a small sample of PsP and TP patients. We found that 97% of PsP patients received RT and that PsP typically occurs within 6 months of definitive therapy. In our select group of PsP and TP patients with available detailed radiation dosimetry data, all PsP and TP areas were found to have received some level of radiation, but no significant difference between the groups was identified. Further, PsP was more common in high-grade gliomas, although further analysis may be necessary to determine if this effect is related to the typically higher RT dose of 60 Gy used for high-grade patients versus 54 Gy for low-grade patients.

Of note, we observed several survival trends in our entire cohort that did not remain significant within each glioma subtype. PsP was found to predict increased OS in all patients but was not significant in the LA and G4 Astro subtypes. Preoperative enhancement predicted decreased OS in all patients but was not significant in any subtype stratification. This possibly reflects that these associations are stronger in certain subtypes or that the association was not sufficiently strong in subtypes with lower sample sizes. Similarly, when examining time to PsP or TP from treatment initiation, PsP was found to occur earlier than TP in all subtypes except G4 Astro. This may be due to the propensity for early disease progression in G4 Astro patients.

Although we have derived a large cohort, the conclusions will need to be validated in additional retrospective cohorts and prospective studies. Additionally, examination of the true incidence of CEnew, PsP, and TP is limited since this study utilized censored data and censored patients could develop CEnew, PsP, and TP later in their clinical course. There is a possibility that CEnew occurred as a result of steroid taper. Further, we utilized strict criteria to determine an instance of CEnew as PsP, where CEnew triggering treatment initiation was deemed as TP. Of note, patients who met radiographic criteria for PsP while maintained on the same active treatment were still placed in the PsP group (Supplementary Figure S9). While these criteria ensured CEnew resolution was not due to treatment response, we have likely underreported the PsP incidence in cases where PsP was interpreted as TP and treated. Seyve et al. estimate this to be ~20%.^6^ Our investigation was also limited by the available patient data, as not all patients were tested for *MGMT* promoter methylation status or CDKN2A loss.

**Figure 4.**
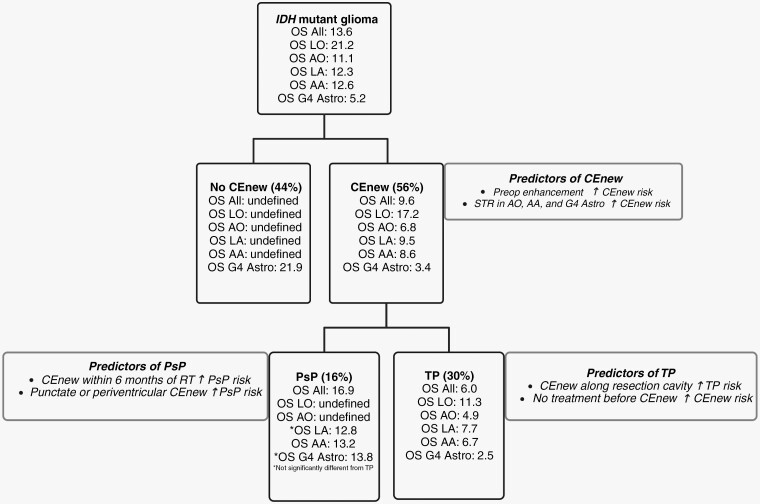
Survival implications and predictors of CEnew, PsP, and TP. Flowchart depicting summary of findings and the change in PsP/TP risk and survival outcomes at various points in the clinical course of an *IDH* mutant glioma patient. Other results not shown here include, PsP duration = 8 months, 11% of PsP does not resolve, and repeat RT did not increase PsP risk. All OS values are in years and percentages are of all patients (*n* = 587, 10% are censored CEnew). CEnew, new contrast enhancement; *IDH*, isocitrate dehydrogenase; OS, overall survival; PsP, pseudoprogression; RT, radiation therapy.

In conclusion, the present study has identified PsP as a common event in *IDH* mutant gliomas that is associated with improved survival relative to TP and similar survival relative to patients without CEnew and one that is characterized by distinct radiographic features and earlier time of onset compared with TP. Notably, we have found that PsP can be reliably differentiated from TP based on the following criteria: CEnew appearance within 6 months of initial definitive therapy, punctate size, and periventricular location. Additionally, PsP should only be considered in patients that have received chemotherapy or RT. Our study serves as the largest analysis of PsP patients and shows PsP has similar characteristics in high- and low-grade *IDH* mutant patients. The incidence, time of onset, and radiographic characteristics of PsP reported here can be utilized by clinicians to aid in the differentiation between PsP and TP when faced with CEnew. Further, our examination of survival in patients with CEnew, PsP, and TP can be used as a guide for clinicians at different bifurcations in a patient’s clinical course (Figure 4).

## Supplementary Material

vdad028_suppl_Supplementary_Material
