# Supplementary material for: Retrospective examination of pseudoprogression in IDH mutant gliomas
Source: Neurooncol Adv. 2023 Mar 18;5(1):vdad028. doi: 10.1093/noajnl/vdad028 (PMC10148681; doi:10.1093/noajnl/vdad028)
Supplement: vdad028_suppl_Supplementary_Material [file vdad028_suppl_supplementary_material.docx]

|  | CEnew (%) | No CEnew (%) | PsP(%) | TP(%) |
| --- | --- | --- | --- | --- |
| LO | 55 (37) | 93 (63) | 16 (17)* | 26 (15)* |
| RT Only/TMZ only/RT + TMZ | 9/10/5 | 7/13/6 | 2/3/2 | 6/7/2 |
| PCV Only/RT + PCV | 2/7 | 2/6 | 0/3 | 2/0 |
| GTR/STR/Biopsy | 22/24/9 | 42/35/16 | 8/6/2 | 11/12/4 |
| *MGMT* Methylated/Unmethylated | 19/5 | 38/13 | 5/1 | 11/2 |
| Bevacizumab | 13 | 1 | 2 | 11 |
| Months from RT/Chemotherapy to CEnew | 38.7 | NA | 18.4 | 45.5 |
| AO | 42 (58) | 31 (42) | 11 (12)* | 25 (14)* |
| RT Only/TMZ only/RT + TMZ | 3/3/19 | 3/3/11 | 0/1/4 | 3/2/15 |
| PCV Only/RT + PCV | 3/13 | 0/2 | 0/7 | 2/4 |
| GTR/STR/Biopsy | 11/30/1 | 15/16 | 1/10/0 | 9/16/0 |
| *MGMT* Methylated/Unmethylated | 16/0 | 9/1 | 7/0 | 6/0 |
| Bevacizumab | 12 | 1 | 1 | 11 |
| Months from RT/Chemotherapy to CEnew | 15.1 | NA | 6.0 | 28.4 |
| LA | 77 (55) | 62 (45) | 22 (24)* | 44 (25)* |
| RT Only/TMZ only/RT + TMZ | 20/5/22 | 5/0/11 | 6/1/9 | 14/4/13 |
| PCV Only/RT + PCV | 0/6 | 0/1 | 0/1 | 0/5 |
| GTR/STR/Biopsy | 22/44/11 | 25/31/6 | 9/10/3 | 10/29/5 |
| *MGMT* Methylated/Unmethylated | 21/23 | 18/23 | 8/5 | 9/16 |
| Bevacizumab | 23 | 3 | 6 | 17 |
| Months from RT/Chemotherapy to CEnew | 15.4 | NA | 5.5 | 15.5 |
| AA | 77 (57) | 58 (43) | 21 (23)* | 39 (22)* |
| RT Only/TMZ only/RT + TMZ | 10/0/62 | 1/0/48 | 1/0/20 | 9/0/42 |
| PCV Only/RT + PCV | 0/3 | 0/1 | 0/1 | 0/2 |
| GTR/STR/Biopsy | 27/42/8 | 33/21/4 | 6/15/0 | 16/17/6 |
| *MGMT* Methylated/Unmethylated | 20/20 | 25/16 | 6/5 | 11/9 |
| Bevacizumab | 31 | 1 | 6 | 25 |
| Months from RT/Chemotherapy to CEnew | 14.0 | NA | 4.3 | 26.1 |
| G4 Astro | 75 (82) | 17 (18) | 22 (24)* | 45 (25)* |
| RT Only/TMZ only/RT + TMZ | 11/0/61 | 0/0/16 | 3/0/19 | 8/0/42 |
| PCV Only/RT + PCV | 0/2 | 0/0 | 0/1 | 0/1 |
| GTR/STR/Biopsy | 28/40/7 | 8/9/0 | 8/13/1 | 19/20/6 |
| *MGMT* Methylated/Unmethylated | 20/13 | 4/5 | 10/3 | 7/8 |
| Bevacizumab | 29 | 1 | 7 | 21 |
| Months from RT/Chemotherapy to CEnew | 9.6 | NA | 4.9 | 11.7 |

**Table S1**: Summary of treatment and *MGMT* methylation status stratified by glioma subtype

| Summary of Results – Patients with Multiple CEnew instances | | | | | | |
| --- | --- | --- | --- | --- | --- | --- |
|  | All | LO | AO | LA | AA | G4 Astro |
| # with Multiple CEnew | 132 (40%) | 24 (16%) | 14 (19%) | 31(22%) | 31 (23%) | 32 (35%) |
| Median Time Between 1^st^ and 2^nd^ CEnew (months) | 13.1 | 18.2 | 14.8 | 13.3 | 8.8 | 8.0 |
| # of Patients w/ 1 CEnew Instance | 193 | 31 | 27 | 46 | 46 | 43 |
| # of Patients w/ 2 CEnew instances | 92 | 15 | 7 | 23 | 26 | 21 |
| # of Patients w/ 3 CEnew instances | 25 | 4 | 5 | 5 | 2 | 9 |
| # of Patients w/ 4 CEnew instances | 11 | 5 | 1 | 3 | 2 | 0 |
| # of Patients w/ 5 CEnew instances | 3 | 0 | 1 | 0 | 1 | 1 |
| # of Patients w/ 6 CEnew instances | 1 | 0 | 0 | 0 | 0 | 1 |
| Concurrent CEnew | 32 | 5 | 5 | 6 | 7 | 9 |
| Serial CEnew | 66 | 11 | 16 | 11 | 16 | 12 |
| Synchronous CEnew | 11 | 4 | 2 | 2 | 2 | 1 |

**Table S2:** Summary of CEnew instances. Concurrent instances of CEnew were defined as those that appeared on separate scans but were both present on subsequent scans. Synchronous instances of CEnew appeared on the same scan. Serial instances of CEnew were defined as those that were never present on the same scan.

**Figure S1**: KM Curve to first CEnew stratified by glioma subtype (LO vs G4 Astro p < 0.0001)

|  | Preop Enhancing (%) | Preop Nonenhancing (%) | Unknown |
| --- | --- | --- | --- |
| All (n = 587) | 153 (26) | 280 (48) | 154 (26) |
| LA (n = 139) | 24 (17) | 86 (62) | 29 (21) |
| AA (n = 135) | 33 (24) | 74 (55) | 28 (21) |
| LO (n = 147) | 18 (12) | 92 (63) | 38 (25) |
| AO (n = 73) | 27 (37) | 19 (26) | 27 (37) |
| G4 Astro (n = 92) | 51 (55) | 9 (10) | 32 (35) |

**Table S3**: Summary of preoperative enhancement status of *IDH* mutant cohort.

| Variable (EFS) | All (n = 433) | | | LO Patients (n = 110) | | | AO Patients (n = 46) | | |
| --- | --- | --- | --- | --- | --- | --- | --- | --- | --- |
|  | HR | p-value | 95% CI | HR | p-value | 95% CI | HR | p-value | 95% CI |
| Age at Intervention | 1.01 | 0.02* | [1.00, 1.03] | 0.99 | 0.9 | [0.95, 1.05] | 1.05 | 0.007* | [1.01, 1.09] |
| Gender (Ref = F) | 0.94 | 0.7 | [0.71, 1.25] | 1.00 | 0.9 | [0.475, 2.11] | 0.36 | 0.03* | [0.14, 0.92] |
| KPS <= 70 | 0.70 | 0.3 | [0.37, 1.33] | NA | NA | NA | 0.02 | 0.002* | [0.001, 0.22] |
| GTR (Ref = STR/Biopsy) | 0.71 | 0.2 | [0.43, 1.13] | 0.71 | 0.4 | [0.33, 1.53] | 0.53 | 0.2 | [0.20, 1.45] |
| Preop Enhancement (Ref = No Preop Enhancement | 1.39 | 0.02* | [1.05, 1.84] | 0.64 | 0.3 | [0.28, 1.45] | 0.68 | 0.4 | [0.25, 1.83] |
| Events | 110 | | | 36 | | | 27 | | |
| Variable (EFS) | LA Patients (n = 110) | | | AA Patients (n = 107) | | | G4 Astro Patients (n = 60) | | |
|  | HR | p-value | 95% CI | HR | p-value | 95% CI | HR | p-value | 95% CI |
| Age at Intervention | 1.06 | 0.002* | [1.02, 1.09] | 1.01 | 0.678 | [0.97, 1.04] | 1.01 | 0.4 | [0.98, 1.04] |
| Gender (F =ref) | 0.52 | 0.04* | [0.28, 0.99] | 0.51 | 0.034 | [0.27, 0.95] | 1.80 | 0.08 | [0.93, 3.47] |
| KPS <= 70 | 1.02 | 0.9 | [0.31, 3.37] | 1.90 | 0.678 | [0.40, 9.21] | 1.13 | 0.9 | [0.31, 4.15] |
| GTR (STR/Biopsy = ref) | 0.55 | 0.07 | [0.29, 1.07] | 0.44 | 0.008* | [0.23, 0.80] | 0.64 | 0.2 | [0.32, 1.27] |
| Preop Enhancement | 0.94 | 0.865 | [0.49, 1.83] | 2.09 | 0.020* | [1.12, 3.88] | 2.08 | 0.2 | [0.66, 6.55] |
| Events | 55 | | | 56 | | | 45 | | |

**Table S4**: Multivariate analysis results with Preop Enhancement covariate as a predictor of enhancement free survival (EFS).

**Figure S2:** Univariate KM analysis of OS of patients with preoperatively enhancing versus nonenhancing tumors. (**A**) All patients(p <0.0001). (**B-F**) separated by LO (p = 0.5), AO (p = 0.05), LA (p = 0.004), AA (p = 0.1), and G4 Astro (p = 0.1) diagnoses, respectively.

| Variable (OS) | All (n = 433) | | | LO Patients (n = 110) | | | AO Patients (n = 46) | | |
| --- | --- | --- | --- | --- | --- | --- | --- | --- | --- |
|  | HR | p-value | 95% CI | HR | p-value | 95% CI | HR | p-value | 95% CI |
| Age at Intervention | 0.99 | 0.9 | [0.98, 1.02] | 1.01 | 0.6 | [0.96, 1.07] | 1.02 | 0.4 | [0.97, 1.07] |
| Gender (Ref = f) | 1.21 | 0.4 | [0.81, 1.81] | 1.90 | 0.2 | [0.70, 5.11] | 0.44 | 0.2 | [0.14, 1.45] |
| KPS <= 70 | 3.91 | 0.0003* | [1.88, 8.11] | NA | NA | NA | 0.87 | 0.9 | [0.09, 9.45] |
| GTR (Ref = STR/Biopsy) | 0.63 | 0.03* | [0.42, 0.95] | 0.51 | 0.2 | [0.18, 1.47] | 0.60 | 0.4 | [0.19, 1.94] |
| Preop Enhancement (Ref = No Preop Enhancement | 2.37 | <0.0001 | [1.61, 3.49] | 0.77 | 0.7 | [0.25, 2.40] | 4.20 | 0.03* | [1.19, 14.8] |
| Events | 110 | | | 19 | | | 17 | | |
| Variable (OS) | LA Patients (n = 110) | | | AA Patients (n = 107) | | | G4 Astro Patients (n = 60) | | |
|  | HR | p-value | 95% CI | HR | p-value | 95% CI | HR | p-value | 95% CI |
| Age at Intervention | 0.99 | 0.5 | [0.95, 1.03] | 0.97 | 0.3 | [0.92, 1.03] | 1.04 | 0.04* | [1.00, 1.08] |
| Gender (F =ref) | 0.67 | 0.4 | [0.24, 1.83] | 1.43 | 0.5 | [0.49, 4.23] | 1.49 | 0.5 | [0.62, 3.55] |
| KPS <= 70 | 8.68 | 0.002* | [2.24, 33.6] | 13.22 | 0.002* | [2.60, 67.2] | 0.79 | 0.6 | [0.34, 7.65] |
| GTR | 0.35 | 0.03* | [0.13, 0.92] | 0.58 | 0.3 | [0.22, 1.50] | 1.34 | 0.5 | [0.57, 3.16] |
| Preop Enhancement (Ref = No Preop Enhancement) | 4.63 | 0.002* | [1.79, 12.0] | 1.76 | 0.2 | [0.70, 4.33] | 2.89 | 0.2 | [0.65, 12.9] |
| Events | 25 | | | 23 | | | 26 | | |

**Table S5:** Multivariate analysis of OS in all patients with known preoperative enhancement status including Preoperative Enhancement covariate to examine its prognostic impact.

| All Patients  p < 0.0001 | No CEnew | Yes CEnew | % with CEnew |
| --- | --- | --- | --- |
| Preop Enhancing (n = 153) | 41 | 112 | 73.2 |
| Preop Nonenhancing (n = 280) | 172 | 108 | 38.5 |
| Total (n = 433) | 213 | 220 | 50.8 |

| LO Patients  p = 0.05 | No CEnew | Yes CEnew | % with CEnew |
| --- | --- | --- | --- |
| Preop Enhancing (n = 18) | 8 | 10 | 55.6 |
| Preop Nonenhancing (n =91 ) | 64 | 27 | 29.6 |
| Total (n = 109 ) | 72 | 37 | 33.9 |

| AO Patients  p = 0.01 | No CEnew | Yes CEnew | % with CEnew |
| --- | --- | --- | --- |
| Preop Enhancing (n = 27) | 7 | 20 | 74.1 |
| Preop Nonenhancing (n = 19) | 12 | 7 | 58 |
| Total (n = 46) | 19 | 27 | 58.7 |

| LA Patients  p = 0.001 | No CEnew | Yes CEnew | % with CEnew |
| --- | --- | --- | --- |
| Preop Enhancing (n = 24) | 5 | 19 | 76.0 |
| Preop Nonenhancing (n = 86) | 50 | 36 | 41.9 |
| Total (n = 110) | 55 | 55 | 50.0 |

| AA Patients  p = 0.009 | No CEnew | Yes CEnew | % with CEnew |
| --- | --- | --- | --- |
| Preop Enhancing (n = 33) | 9 | 24 | 72.7 |
| Preop Nonenhancing (n = 73) | 40 | 33 | 45.2 |
| Total (n = 106) | 49 | 57 | 53.8 |

| G4 Astro Patients  p = 0.07 | No CEnew | Yes CEnew | % with CEnew |
| --- | --- | --- | --- |
| Preop Enhancing (n = 51) | 12 | 39 | 76.5 |
| Preop Nonenhancing (n = 9) | 3 | 6 | 66.7 |
| Total (n = 60) | 15 | 45 | 75.0 |

**Table S6**: Two-way table examining preoperative enhancement and the development of CEnew

| Variable (OS) | All (n= 587) | | | LO CEnew vs No CEnew (n = 148) | | | AO CEnew vs No CEnew (n = 73) | | |
| --- | --- | --- | --- | --- | --- | --- | --- | --- | --- |
|  | HR | p-value | 95% CI | HR | p-value | 95% CI | HR | p-value | 95% CI |
| Age at Intervention | 1.01 | 0.03* | [1.00, 1.03] | 1.01 | 0.7 | [0.97, 1.04] | 1.02 | 0.2 | [0.99, 1.06] |
| Gender (F =ref) | 0.93 | 0.6 | [0.69, 1.25] | 1.24 | 0.6 | [0.56, 2.72] | 0.51 | 0.1 | [0.23, 1.14] |
| KPS <= 70 | 1.72 | 0.03* | [1.06, 2.79] | 1.28 | 0.8 | [0.16, 9.98] | 1.15 | 0.8 | [0.37, 3.60] |
| GTR | 0.81 | 0.2 | [0.59, 1.10] | 0.44 | 0.04* | [0.20, 0.96] | 1.10 | 0.8 | [0.49, 2.46] |
| CEnew | 5.28 | <0.0001* | [3.45, 8.10 | 3.57 | 0.004* | [1.51, 8.42] | 3.83 | 0.004* | [1.52, 9.68] |
| Events | 193 | | | 31 | | | 31 | | |
| Variable (OS) | LA CEnew vs No CEnew (n = 139) | | | AA CEnew vs No CEnew (n = 135) | | | G4 Astro CEnew vs No CEnew (n = 92) | | |
|  | HR | p-value | 95% CI | HR | p-value | 95% CI | HR | p-value | 95% CI |
| Age at Intervention | 0.99 | 0.6 | [0.96, 1.02] | 1.00 | 0.9 | [0.96, 1.05] | 1.04 | 0.0009* | [1.02, 1.07] |
| Gender (F =ref) | 0.73 | 0.4 | [0.37, 1.43] | 0.73 | 0.4 | [0.37, 1.47] | 1.48 | 0.2 | [0.79, 2.78] |
| KPS <= 70 | 0.73 | 0.6 | [0.24, 2.21] | 2.58 | <0.0001* | [1.52, 5.87] | 1.49 | 0.4 | [0.59, 3.74] |
| GTR | 0.45 | 0.04* | [0.21, 0.97] | 0.94 | 0.9 | [0.48, 1.85] | 1.59 | 0.1 | [0.87, 2.90] |
| CEnew | 5.69 | 0.0009* | [2.02, 16.02] | 9.18 | <0.0001* | [3.15, 26.74] | 3.33 | 0.05* | [0.99, 11.1] |
| Events | 42 | | | 40 | | | 49 | | |

**Table S7:** Multivariate analysis of OS by glioma subtype including CEnew covariate to examine prognostic impact of CEnew

| Patients with Treatment Prior to CEnew, PsP, or TP | | | |
| --- | --- | --- | --- |
|  | % with RT prior to Appearance | % with Chemotherapy Alone Prior to Appearance | % with Spontaneous Appearance |
| CEnew - All | 82% (223/271) | 7% (19/271) | 11% (29/271) |
| CEnew - LO | 67% (29/43) | 21%( 9/43) | 12% (5/43) |
| CEnew – AO | 83% (30/36) | 3% (1/36) | 14% (5/36) |
| CEnew – LA | 73% (48/66) | 9% (6/66) | 18% (12/66) |
| CEnew – AA | 95% (56/59) | 3% (2/59) | 1% (1/59) |
| CEnew – G4 Astro | 98% (65/67) | 1% (1/67) | 1% (1/67) |
| PsP - All | 97% (89/92) | 3% (3/92) | 0% (0/92) |
| PsP - LO | 88%( 14/16) | 12% (2/16) | 0% (0/16) |
| PsP - AO | 100% (11/11) | 0% (0/11) | 0% (0/11) |
| PsP - LA | 100% (22/22) | 0% (0/22) | 0% (0/22) |
| PsP - AA | 100% (21/21) | 0% (0/21) | 0% (0/21) |
| PsP – G4 Astro | 95% (21/22) | 5% (1/22) | 0% (0/22) |
| TP - All | 75% (134/179) | 9% (16/179 ) | 16% (29/179) |
| TP – LO | 44% (15/27) | 26% (7/27) | 19% (5/27) |
| TP – AO | 76% (19/25) | 4% (1/25) | 20% (5/25) |
| TP – LA | 59% (26/44) | 14% (6/44) | 27% (12/44) |
| TP – AA | 92% (35/38) | 5% (2/38) | 3% (1/38) |
| TP – G4 Astro | 98% (44/45) | 0% (0/45) | 2% (1/45) |

**Table S8**: Summary of treatments patients received before developing CEnew, PsP, and TP.

| Variable (Time to PsP) | All (n = 445) | | | LO Patients (n = 79) | | | AO Patients (n = 57) | | |
| --- | --- | --- | --- | --- | --- | --- | --- | --- | --- |
|  | HR | p-value | 95% CI | HR | p-value | 95% CI | HR | p-value | 95% CI |
| Age at Intervention | 1.03 | 4.0e-8* | [1.02, 1.03] | 1.01 | 0.3 | [0.99, 1.03] | 1.02 | 0.2 | [0.99, 1.05] |
| Gender (Ref = F) | 0.90 | 0.3 | [0.74, 1.09] | 1.09 | 0.2 | [0.68, 1.78] | 0.48 | 0.02* | [0.26, 0.91] |
| KPS <= 70 | 1.35 | 0.1 | [0.94, 1.92] | 3.30 | 0.01* | [1.27, 8.57] | 0.78 | 0.6 | [0.31, 1.97] |
| GTR (Ref = STR/Biopsy) | 0.57 | <0.0001* | [0.47, 0.70] | 0.63 | 0.06 | [0.39, 1.02] | 0.60 | 0.2 | [0.30, 1.21] |
| PsP (Ref. TP) | 1.29 | 0.02* | [1.04, 1.61] | 1.01 | 0.9 | [0.56, 1.81] | 2.69 | 0.02* | [1.24, 5.83] |
| Events | 445 | | | 79 | | | 57 | | |
| Variable (Time to PsP) | LA Patients (n = 102) | | | AA Patients (n = 99) | | | G4 Astro Patients (n = 107) | | |
|  | HR | p-value | 95% CI | HR | p-value | 95% CI | HR | p-value | 95% CI |
| Age at Intervention | 1.05 | 0.002* | [1.02, 1.07] | 1.01 | 0.7 | [0.98, 1.04] | 1.03 | 0.002* | [1.01, 1.05] |
| Gender (Ref = F) | 0.97 | 0.9 | [0.64, 1.47] | 0.93 | 0.8 | [0.60, 1.46] | 0.93 | 0.8 | [0.62, 1.41] |
| KPS <= 70 | 1.48 | 0.2 | [0.77, 2.83] | 1.49 | 0.34 | [0.65, 3.41] | 0.99 | 0.9 | [0.34, 2.91] |
| GTR (Ref = STR/Biopsy) | 0.41 | 0.0002* | [0.26, 0.65] | 0.45 | 0.002* | [0.27, 0.74] | 1.04 | 0.9 | [0.68, 1.58] |
| PsP (Ref = TP) | 1.69 | 0.04* | [1.03, 2.77] | 2.24 | 0.003* | [1.31, 3.84] | 0.93 | 0.8 | [0.62, 1.41] |
| Events | 102 | | | 99 | | | 107 | | |

**Table S9**: Multivariate analysis of time to PsP and TP using PsP covariate.

**Figure S3:** Kaplan-Meier analysis examining the time from initial surgery to PsP or TP for All (**A**), LO (**B**), AO (**C**), LA (**D**), AA (**E**), and GBM (**F**) and duration of PsP (**G-H**). (**A-F**) show time to PsP and time to TP. Log-rank P values comparing time to PsP versus TP in All, LO, AO, LA, AA, and GBM were 0.0005, 0.8, 0.0002, 0.03, <0.0001, and 0.3, respectively. (**G-H**) show duration of all PsP instances and PsP duration by glioma subtype (Log-rank P = 0.17).

| Variable (PsP Duration) | Duration of PsP (n=92) | | |
| --- | --- | --- | --- |
|  | HR | p-value | 95% CI |
| Age at Intervention | 0.99 | 0.9 | [0.96, 1.04] |
| KPS <= 70 | 3.89 | 0.1 | [0.64, 23.61] |
| Gender (F =ref) | 2.09 | 0.07 | [0.95, 4.60] |
| GTR | 2.88 | 0.1 | [1.29, 6.43] |
| Dx; WHO 2016. |  |  |  |
| LO | 1.0 | -- | -- |
| AO | 0.76 | 0.7 | [0.21, 2.77] |
| LA | 0.54 | 0.3 | [0.19, 1.54] |
| AA | 1.29 | 0.7 | [0.41, 4.04] |
| G4 Astro | 0.80 | 0.7 | [0.26, 2.43] |
| Events | 92 | | |

**Table S10**: Multivariate analysis of PsP duration in all PsP patients.

**Figure S4:** Univariate KM analysis of OS of patients with PsP vs patients with TP. (**A**) OS of all patients with PsP versus all with TP (p <0.0001). (**B-F**) OS of patients with PsP versus patients with TP separated by LO (p = 0.0003), AO (p = 0.008), LA (p = 0.04), AA (p = 0.001), and G4 Astro (p < 0.0001) diagnoses, respectively.

| Variable (OS) | All (Ref. LO) (n=587) | | | LO + AO (n=221) | | | LA (n = 139 ) | | |
| --- | --- | --- | --- | --- | --- | --- | --- | --- | --- |
|  | HR | p-value | 95% CI | HR | p-value | 95% CI | HR | p-value | 95% CI |
| Age at Intervention | 1.03 | <0.0001* | [1.02, 1.05] | 1.04 | 0.007* | [1.01, 1.07] | 1.03 | 0.2 | [0.99, 1.06] |
| Gender (Ref = F) | 0.85 | 0.3 | [0.61, 1.17] | 0.78 | 0.4 | [0.41, 1.46] | 0.86 | 0.7 | [0.41, 1.78] |
| KPS <= 70 | 1.07 | 0.8 | [0.62, 1.85] | 0.85 | 0.8 | [0.23, 3.10] | 0.40 | 0.2 | [0.11, 1.48] |
| GTR (Ref = STR/Biopsy) | 1.01 | 0.9 | [0.72, 1.42] | 0.91 | 0.8 | [0.48, 1.72] | 0.23 | 0.006* | [0.08, 0.66] |
| PsP | 0.42 | 0.001* | [0.25, 0.71] | 0.15 | 0.01* | [0.04, 0.65] | 1.94 | 0.3 | [0.62, 6.10] |
| TP | 4.18 | 0.002* | [1.72, 10.14] | 3.98 | 0.03* | [3.56, 4.53 | 17.6 | 0.002* | [2.78, 110.5] |
| Events | 193 | | | 62 | | | 42 | | |
| Variable (OS) | AA (n = 135) | | | LA + AA (n =274) | | | G4 Astro (n = 92) | | |
|  | HR | p-value | 95% CI | HR | p-value | 95% CI | HR | p-value | 95% CI |
| Age at Intervention | 0.99 | 0.8 | [0.94, 1.05] | 1.01 | 0.7 | [0.98, 1.03] | 1.03 | 0.03* | [1.00, 1.01] |
| Gender (Ref = F) | 1.48 | 0.4 | [0.57, 3.84] | 0.88 | 0.6 | [0.52, 1.49] | 0.74 | 0.4 | [0.37, 1.51] |
| KPS <= 70 | 2.2 | 0.0001* | [4.57, 109.1] | 0.99 | 0.9 | [0.40, 2.49] | 1.3 | 0.6 | [0.52, 3.29] |
| GTR (Ref = STR/Biopsy) | 1.60 | 0.3 | [0.71, 3.58] | 0.77 | 0.4 | [0.44, 1.35] | 1.88 | 0.07 | [0.95, 3.71] |
| PsP | 0.19 | 0.01* | [0.05, 0.72] | 0.56 | 0.1 | [0.26, 1.21] | 0.45 | 0.1 | [0.17, 1.25] |
| TP | 0.67 | 0.7 | [0.9, 4.77] | 2.97 | 0.08 | [0.87, 10.2] | 6.31 | 0.03* | [1.18, 33.68] |
| Events | 40 | | | 82 | | | 49 | | |

**Table S11:** Multivariate analysis of OS by glioma subtype including PsP and TP covariates to examine prognostic impact of PsP and TP. PsP and TP covariates are referenced against patient’s without PsP or TP, as patients could have had PsP alone, TP alone, both PsP and TP, or neither PsP or TP.

**Figure S5:** Univariate KM analysis of residual OS in patients with TP or PsP. (**A**) All patients (p < 0.0001). (**B-F**) separated by LO (p = 0.01), AO (p = 0.02), LA (p = 0.003), AA (p = 0.003), and G4 Astro (p < 0.0001) diagnoses, respectively.

| Variable (resOS) | All (Ref. LO) (n = 272) | | | LO Patients (n = 43) | | | AO Patients (n = 36) | | |
| --- | --- | --- | --- | --- | --- | --- | --- | --- | --- |
|  | HR | p-value | 95% CI | HR | p-value | 95% CI | HR | p-value | 95% CI |
| Age at Intervention | 1.01 | 0.2 | [0.99, 1.02] | 1.00 | 0.8 | [0.96, 1.04] | 1.04 | 0.1 | [0.99, 1.08] |
| Gender (Ref = F) | 0.73 | 0.06 | [0.53, 1.01] | 0.84 | 0.7 | [0.34, 2.08] | 0.40 | 0.06 | [0.15, 1.04] |
| KPS <= 70 | 1.82 | 0.03* | [1.07, 3.08] | 2.24 | 0.5 | [0.26, 19.5] | 2.77 | 0.1 | [0.71, 10.80] |
| GTR (Ref = STR/Biopsy) | 1.27 | 0.2 | [0.91, 1.78] | 0.81 | 0.6 | [0.34, 2.08] | 1.43 | 0.4 | [0.59, 3.47] |
| PsP (Ref. TP) | 0.17 | <0.0001* | [0.11, 2.28] | 0.05 | 0.005* | [0.007, 0.42] | 0.07 | 0.01* | [0.008, 0.52] |
| Events | 156 | | | 22 | | | 23 | | |
| Variable (resOS) | LA Patients (n = 66) | | | AA Patients (n = 60) | | | G4 Astro Patients (n = 67) | | |
|  | HR | p-value | 95% CI | HR | p-value | 95% CI | HR | p-value | 95% CI |
| Age at Intervention | 0.97 | 0.1 | [0.93, 1.01] | 0.99 | 0.9 | [0.96, 1.04] | 1.01 | 0.3 | [0.98, 1.04] |
| Gender (Ref = F) | 0.84 | 0.6 | [0.41, 1.73] | 0.86 | 0.7 | [0.39, 1.88] | 0.48 | 0.05* | [0.23, 1.00] |
| KPS <= 70 | 1.77 | 0.3 | [0.59, 5.30] | 10.3 | 0.002* | [2.27, 46.7] | 0.97 | 0.95 | [0.39, 2.43] |
| GTR (Ref = STR/Biopsy) | 0.65 | 0.3 | [0.27, 1.58] | 1.69 | 0.2 | [0.74, 3.85] | 1.96 | 0.5 | [0.97, 3.86] |
| PsP (Ref. TP) | 0.32 | 0.007* | [0.14, 0.73] | 0.23 | 0.004* | [0.08, 0.62] | 0.16 | <0.0001* | [0.07, 0.39] |
| Events | 37 | | | 32 | | | 42 | | |

**Table S12**: Multivariate analysis of resOS by glioma subtype

**Figure S6**: KM curves of OS of PsP versus No CEnew patients. (**A**) All patients (p = 0.01). (**B-F**) separated by LO (p = 0.5, AO (p = 0.9), LA (p = 0.02), AA (p = 0.1), and G4 Astro (p = 0.4) diagnoses, respectively.

| Variable (OS) | All (Ref. LO) (n = 353) | | | LO Patients (n = 109) | | | AO Patients (n = 42) | | | |
| --- | --- | --- | --- | --- | --- | --- | --- | --- | --- | --- |
|  | HR | p-value | 95% CI | HR | p-value | 95% CI | HR | p-value | 95% CI | |
| Age at Intervention | 1.02 | 0.2 | [0.99, 1.04] | 1.04 | 0.2 | [0.97, 1.11] | 9.59 | 0.3 | [0.88, 1.03] | |
| Gender (Ref = F) | 0.88 | 0.7 | [0.50, 1.55] | 2.11 | 0.3 | [0.50, 8.85] | 5.19 | 0.5 | [0.09, 3.12] | |
| KPS <= 70 | 3.3 | 0.05* | [1.01, 11.02] | NA | NA | NA | NA | NA | NA | |
| GTR (Ref = STR/Biopsy) | 0.82 | 0.5 | [0.46, 1.48] | 5.65 | 0.4 | [[0.13, 2.53] | 1.94 | 0.1 | [0.02, 1.73] | |
| PsP (ref = No CEnew) | 1.99 | 0.02* | [1.13, 3.53] | 6.19 | 0.6 | [0.11, 3.38] | 5.70 | 0.6 | [0.06, 5.18] | |
| Events | 49 | | | 9 | | | 7 | | | |
| Variable (OS) | LA Patients (n = 84) | | | AA Patients (n = 79) | | | G4 Astro Patients (n = 39) | | | |
|  | HR | p-value | 95% CI | HR | p-value | 95% CI | HR | p-value | 95% CI | |
| Age at Intervention | 1.01 | 0.8 | [0.95, 1.07] | 1.03 | 0.3 | [0.97, 1.01] | 1.06 | 0.05* | [0.99, 1.13] | |
| Gender (Ref = F) | 0.63 | 0.3 | [0.16, 1.76] | 0.68 | 0.6 | [0.15, 3.05] | 1.87 | 0.4 | [0.44, 7.86] | |
| KPS <= 70 | 5.88 | 0.06 | [0.95, 36.4] | 2.4 | 0.01* | [1.68, 4.44] | NA | NA | NA | |
| GTR (Ref = STR/Biopsy) | 1.06 | 0.9 | [0.28, 3.94] | 0.71 | 0.7 | [0.16, 3.22] | 2.15 | 0.3 | [0.53, 8.66] | |
| PsP (ref = No CEnew) | 5.44 | 0.01* | [1.35, 21.9 | 4.06 | 0.1 | [0.83, 19.75] | 1.36 | 0.7 | | [0.33, 5.64] |
| Events | 12 | | | 9 | | | 12 | | | |

**Table S13:** Multivariate analysis of patients with PsP and No CEnew

| Variable (OS) | All (Ref. LO) (n = 317) | | | LO Patients (n = 102) | | | AO Patients (n = 40) | | | |
| --- | --- | --- | --- | --- | --- | --- | --- | --- | --- | --- |
|  | HR | p-value | 95% CI | HR | p-value | 95% CI | HR | p-value | 95% CI | |
| Age at Intervention | 1.03 | 0.03* | [1.00, 1.07] | 1.04 | 0.3 | [0.96, 1.12] | 9.60 | 0.3 | [0.89, 1.03] | |
| Gender (Ref = F) | 0.93 | 0.8 | [0.46, 1.87] | 2.44 | 0.3 | [0.47, 12.80] | 5.20 | 0.5 | [0.086, 3.12] | |
| KPS <= 70 | 5.00 | 0.01* | [1.42, 17.5] | NA | NA | NA | NA | NA | NA | |
| GTR (Ref = STR/Biopsy) | 0.55 | 0.1 | [0.25, 1.19] | 6.19 | 0.6 | [0.12, 3.21] | 1.92 | 0.1 | [0.02, 1.71] | |
| PsP (ref = No CEnew) | 0.79 | 0.6 | [0.32, 1.91] | 3.04 | 1.00 | [0.00, 100.0] | 1.98 | 0.7 | [0.14, 22.1] | |
| Events | 32 | | | 7 | | | 7 | | | |
| Variable (OS) | LA Patients (n = 75) | | | AA Patients (n = 70) | | | G4 Astro Patients (n = 30) | | | |
|  | HR | p-value | 95% CI | HR | p-value | 95% CI | HR | p-value | 95% CI | |
| Age at Intervention | 1.03 | 0.5 | [0.95, 1.12] | 1.08 | 0.1 | [0.98, 1.19] | 1.23 | 0.02* | [1.03, 1.49] | |
| Gender (Ref = F) | 0.76 | 0.8 | [0.13, 4.63] | 0.39 | 0.4 | [0.05, 3.05] | 8.15 | 0.9 | [0.06, 10.4] | |
| KPS <= 70 | 6.97 | 0.05* | [0.99, 48.9] | 14.5 | 0.04* | [1.01, 208.6] | NA | NA | NA | |
| GTR (Ref = STR/Biopsy) | 0.53 | 0.6 | [0.06, 5.26] | 0.12 | 0.2 | [0.005, 2.67] | 1.33 | 0.8 | 0.10, 18.48] | |
| PsP (ref = No CEnew) | 1.33 | 0.8 | [0.14, 12.2] | 2.18 | 0.5 | [0.29, 16.45] | 5.89 | 0.2 | | [0.001, 1.81= |
| Events | 6 | | | 6 | | | 6 | | | |

**Table S14:** Multivariate analysis of the No CEnew group and PsP patients who did not also have TP.

**Figure S7:** Univariate KM analysis of patients with no CEnew versus PsP, where patients with both PsP and TP have been removed from the PsP group. p values are comparing the “PsP” and “No CEnew” strata. (**A**) OS of all patients with PsP versus all with No CEnew (p = 0.9). (**B-F**) OS of patients with PsP versus patients with no CEnew separated by LO (p = 0.1), AO (p = 0.4), LA (p = 0.6), AA (p = 0.4), and G4 Astro (p = 0.8 diagnoses, respectively.

|  | All | PsP(%) | TP(%) |
| --- | --- | --- | --- |
| Periventricular | 27 | 23 (85)* | 4 (15) |
| Resection Margin | 339 | 41 (12) | 298 (88)* |
| Band Thickening | 48 | 11 (23) | 37 (77) |
| Punctate | 34 | 30 (88)* | 4 (12) |
| Nodular | 283 | 49 (17) | 234 (83) |
| Diffuse Enhancement | 58 | 15 (26) | 43 (74) |
| Synchronous | 11 | 4 (36) | 7 (64) |
| Serial | 49 | 4 (8) | 45 (92) |
| Concurrent | 24 | 9 (38) | 15 (62) |

**Table S15:** Table summarizing the radiographic characteristics and locations of PsP and TP. Asterisks indicate significant difference between PsP and TP (p < 0.05 by Fisher’s Exact Test).

| LO | All | PsP*(%) | TP*(%) |
| --- | --- | --- | --- |
| Periventricular | 6 | 5 (83) | 1 (17) |
| Resection Margin | 58 | 4 (7) | 54 (93) |
| Band Thickening | 8 | 2 (25) | 6 (75) |
| Punctate | 6 | 4 (67) | 2 (33) |
| Nodular | 52 | 4 (8) | 48 (92) |
| Diffuse Enhancement | 4 | 2 (50) | 2 (50) |

| AO | All | PsP*(%) | TP*(%) |
| --- | --- | --- | --- |
| Periventricular | 3 | 3 (100) | 0 |
| Resection Margin | 47 | 6 (13) | 41 (87) |
| Band Thickening | 6 | 2 (33) | 4 (67) |
| Punctate | 3 | 3 (100) | 0 |
| Nodular | 38 | 4 (11) | 34 (89) |
| Diffuse Enhancement | 6 | 2 (33) | 4 (67) |

| LA | All | PsP*(%) | TP*(%) |
| --- | --- | --- | --- |
| Periventricular | 3 | 3 (100) | 0 |
| Resection Margin | 81 | 10 (12) | 71 (88) |
| Band Thickening | 9 | 3 (33) | 6 (67) |
| Punctate | 6 | 6 (100) | 0 |
| Nodular | 67 | 10 (15) | 57 (85) |
| Diffuse Enhancement | 15 | 3 (20) | 12 (80) |

| AA | All | PsP*(%) | TP*(%) |
| --- | --- | --- | --- |
| Periventricular | 6 | 4 (67) | 2 (33) |
| Resection Margin | 74 | 11 (15) | 63 (85) |
| Band Thickening | 7 | 0 | 7 (100) |
| Punctate | 11 | 10 (91) | 1 (9) |
| Nodular | 63 | 14 (22) | 49 (78) |
| Diffuse Enhancement | 15 | 2 (13) | 13 (87) |

| G4 Astro | All | PsP*(%) | TP*(%) |
| --- | --- | --- | --- |
| Periventricular | 10 | 8 (80) | 2 (20) |
| Resection Margin | 78 | 10 (13) | 68 (87) |
| Band Thickening | 18 | 4 (22) | 14 (78) |
| Punctate | 8 | 7 (88) | 1 (12) |
| Nodular | 63 | 17 (27) | 46 (73) |
| Diffuse Enhancement | 17 | 5 (29) | 12 (71) |

**Table S16:** Table summarizing the radiographic features of PsP and TP by subtypes

**Figure S8:** KM curves depicting the OS of PsP patients versus patients with limited follow up at (**A**) 3-, (**B**) 6-, (**C**) 9-, and (**D**) 12-month cutoffs, respectively, for radiographically stable follow up.

**Figure S9:** Univariate KM curve of PsP patients that received treatment (Treated PsP) versus PsP patients that did not receive treatment (True PsP) (p = 0.5).

| Patient | CEnew Type | Prescribed Dose | CEnew completely contained inside PTV? | Mean Dose to Area of CEnew (Gy) |
| --- | --- | --- | --- | --- |
| 1 | PsP | 54 Gy/30 fractions | Yes | 55.34 |
| 2 |  | 54 Gy/30 fractions | No | 46.49 |
| 3 |  | 60 Gy/30 fractions | Yes | 63.12 |
| 4 |  | 60 Gy/30 fractions | No | 27.31 |
| 5 |  | 60 Gy/30 fractions | Yes | 61.35 |
| 6 |  | 60 Gy/30 fractions | Yes | 61.41 |
| 7 |  | 60 Gy/30 fractions | Yes | 63.09 |
| 8 |  | 60 Gy/30 fractions | Yes | 62.71 |
| 9 |  | 60 Gy/30 fractions | Yes | 56.57 |
| 10 |  | 54 Gy/27 fractions | Yes | 56.36 |
| 11 | TP | 60 Gy/30 fractions | Yes | 63.26 |
| 12 |  | 60 Gy/30 fractions | Yes | 62.82 |
| 13 |  | 54 Gy/30 fractions | Yes | 55.74 |
| 14 |  | 60 Gy/30 fractions | Yes | 63.04 |
| 15 |  | 60 Gy/30 fractions | Yes | 60.82 |
| 16 |  | 54 Gy/30 fractions | No | 43.25 |
| 17 |  | 54 Gy/30 fractions | Yes | 56.36 |
| 18 |  | 60 Gy/30 fractions | No | 60.77 |
| 19 |  | 60 Gy/30 fractions | Yes | 47.34 |
| 20 |  | 60 Gy/30 fractions | No | 61.03 |

**Table S17**: Radiation dosimetry data of 20 patients with PsP or TP. PTV = planning target volume
